# The immune response behind peptide vaccination in diffuse midline glioma

**DOI:** 10.1002/1878-0261.13686

**Published:** 2024-06-16

**Authors:** Craig A. Vincent, Silvia Remeseiro

**Affiliations:** ^1^ Department of Medical and Translational Biology, Section of Molecular Medicine Umeå University Sweden; ^2^ Wallenberg Centre for Molecular Medicine (WCMM) Umeå University Sweden

**Keywords:** cancer, diffuse midline glioma, H3K27M mutation, immunotherapy, neoepitope peptide vaccine

## Abstract

A first‐in‐human trial demonstrated that a vaccine targeting the histone mutation H3K27M can induce an immune response, in a mutation‐specific manner, in patients with diffuse midline glioma. In a recent study by Boschert *et al*., the same group now dissects the functional immune response triggered after effective vaccination of one of the patients, who has been in remission for over 3 years. The H3K27M peptide vaccine, named H3‐vac, induces a CD4^+^ T‐cell‐specific immune response in this patient and expands the repertoire of polyclonal H3K27M‐specific T‐cell receptors. A clonal H3K27M‐reactive B‐cell population was also detected in the patient's cerebrospinal fluid. Importantly, the immune response is induced across various human leukocyte antigen alleleotypes, indicating the potential efficacy of the vaccine in diverse populations. By exploring in detail the immune response linked to this patient's long‐term survival, the authors prove peptide vaccinations as a viable therapeutic approach. This paves the way for personalised therapies harnessing immunogenic T‐ and B‐cell responses against different tumour types.

## Graphical abstract

The robust immune response of a diffuse midline glioma (DMG) patient in complete remission for over 3 years after receiving a H3K27M peptide vaccine. CD4^+^ T cells are crucial drivers of the immune response triggered by the H3K27M peptide vaccine. The immune response stimulates H3K27M‐specific B cells, expands the repertoire of polyclonal H3K27M‐specific T‐cell receptors (TCRs) and is induced across diverse human leukocyte antigen (HLA) types. (Created with BioRender.com)

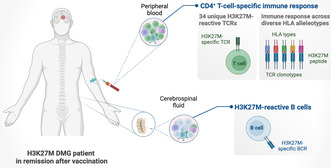

**Abbreviations**
BCR: B‐cell receptor
CSF: cerebrospinal fluid
DMG: diffuse midline glioma
HLA: human leukocyte antigen
PBMC: peripheral blood mononuclear cell
TCR: T‐cell receptor

Vaccines that induce a targeted immune response specifically against cancer cells offer an exciting and encouraging avenue for personalised cancer treatment. Defective proteins, encoded by tumour‐specific mutations, are transported to the cell surface by human leukocyte antigen (HLA) complexes, where they are recognised by T cells, thereby triggering an immune response. However, only a small fraction of these proteins—so‐called neoepitopes—are able to stimulate the immune response spontaneously. Additionally, cancer cells often evolve resistance to this mechanism. Clinical evidence shows that vaccination with neoepitopes can elicit a more robust T‐cell response and thus enhance the innate immune response against tumour cells. While recent studies have highlighted the positive clinical effect of neoepitope vaccines on tumour regression and overall patient survival [1, 2, 3], the intricacies of this immune response had not been well‐characterised.

Boschert et al. [4] dig deeper into the immunological response of a patient who received a tailored H3K27M neoepitope vaccine following a diagnosis of diffuse midline glioma (DMG), a tumour of the central nervous system. Due to its infiltrative nature within midbrain structures and its inherent resistance to standard therapies, DMG poses a significant treatment challenge. A particularly aggressive DMG subtype is characterised by substitution of lysine 27 to methionine in histone H3 (H3K27M). H3K27M DMG patients experience a highly aggressive disease with low survival rates [5]. Given the limited efficacy of current treatments, the H3K27M mutation serves as an attractive target for personalised neoepitope vaccination. In a first‐in‐human clinical trial, the same team had shown that a vaccine targeting H3K27M was not only safe and well‐tolerated, but also five of eight patients receiving the H3K27M_14–40_ peptide vaccine displayed CD4^+^ T‐cell mutation‐specific responses [6]. Importantly, one of the patients sustained complete remission for over 3 years, which provided Boschert et al. with a unique opportunity to characterise the functional immune response in this specific case (Fig. 1).

**Fig. 1:**
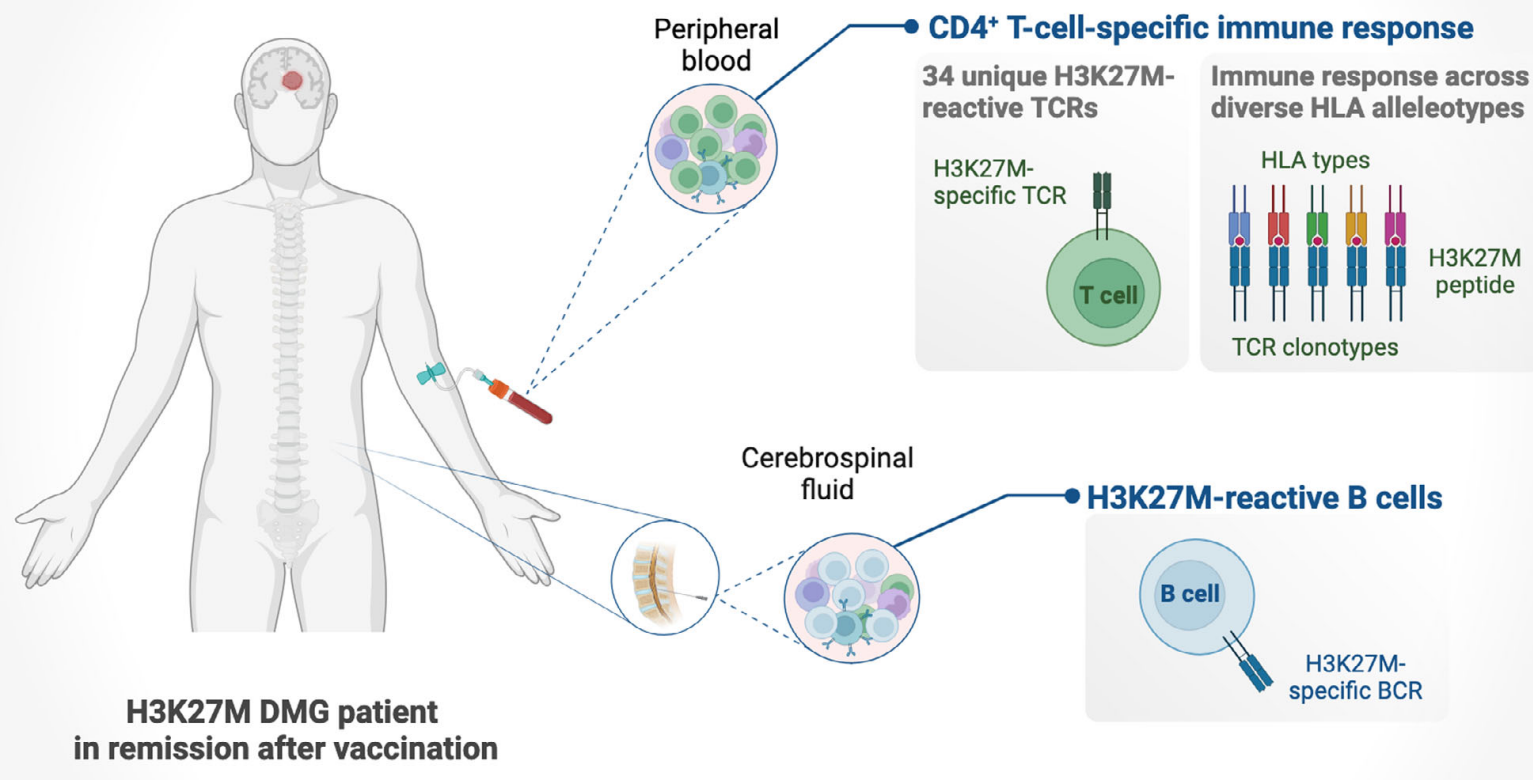
Robust immune response of a diffuse midline glioma (DMG) patient in complete remission for over 3 years after receiving a H3K27M peptide vaccine. CD4^+^ T cells are crucial drivers of the immune response triggered by the H3K27M peptide vaccine. The immune response stimulates H3K27M‐specific B cells, expands the repertoire of polyclonal H3K27M‐specific TCRs and is induced across diverse human leukocyte antigen (HLA) types. (Created with BioRender.com)

To identify the most active immune cells in this patient, the authors isolated peripheral blood mononuclear cells (PBMCs) from blood samples taken at regular intervals during and after treatment with H3‐vac, their H3K27M peptide vaccine. At 4 weeks postvaccination, when the patient demonstrated a strong vaccine‐induced immune response, they observed an enrichment of IFN‐γ secreting‐cells in samples that had been expanded *ex vivo* with the H3K27M peptide and found that these were predominantly CD4^+^ T cells. Furthermore, the authors noted a significant shift in CD4^+^ T‐cell receptor (TCR) clonotype frequencies following peptide stimulation, while CD8^+^ T‐cell clonotypes remained unchanged. This confirms that the vaccine‐induced immune response was primarily driven by CD4^+^ T cells. This discovery aligns with other evidence suggesting that CD4^+^ T cells are as important as CD8^+^ cytotoxic T cells for tumour dissemination [7, 8], and how the activation of CD4^+^ T cells can influence the fitness of CD8^+^ T cells [9]. These findings shed light on why the patient in this study responded exceptionally well to the treatment, underscoring the role of CD4^+^ T cells as the key contributors to an effective targeted vaccine response.

Having observed this robust CD4^+^ T‐cell response, the authors sought to determine the proportion of TCRs specific to the H3K27M epitope, which would indicate specific stimulation by the vaccine. To do so, they first identified 102 clonal TCRs, and used these sequences to establish cell lines expressing each TCR of interest in combination with an integrated GFP reporter gene [10]. These cell lines were then exposed to H3K27M peptides, activating GFP upon TCR:peptide interaction, which demonstrated that 34 of the 102 TCRs specifically reacted to the H3K27M mutant peptide, but not the H3 wt control. Identifying these functionally validated tumour‐specific TCRs enabled the authors to perform a more focused longitudinal follow‐up. During this analysis, they observed that reactive TCR levels remained elevated compared to baseline even at 62 weeks postvaccination, with a peak between weeks 10 and 22, providing insights into the dynamics of the immune response. Interestingly, during this peak period, a particular set of clonally convergent TCRs were the most abundant. Clonally convergent TCRs—those with shared antigen specificity and identical amino acid sequence but unique nucleotide sequences—could potentially serve as biomarkers for favourable response to cancer immunotherapy. Previous research examining data from patients diagnosed with various cancer types that had received immune checkpoint inhibitors had suggested significant association between TCR convergence level and progression‐free survival [11]. The detection of convergent TCRs in the patient described by Boschert et al. corroborates these findings and highlights this as an interesting avenue for further investigation.

In addition to analysing PBMCs, Boschert et al. also examined the cerebrospinal fluid (CSF) of this vaccinated H3K27M patient and identified a subset of H3K27M‐reactive TCRs as central memory CD4^+^ T cells. Recent evidence indicates a superior ability of memory T cells to control tumour growth compared to effector T cells due to their increased persistence and cytokine secretion [12]. This observation is intriguing since memory T cells remain elevated long after immunotherapy treatment, suggesting that the long‐term efficacy of neoepitope vaccine could depend on memory T‐cell responses.

The authors also noted an increase in memory B cell and plasmablast populations in the CSF when compared to publicly available control data sets. While analysing the B‐cell receptor (BCR) sequences of these cells, they found that the two most prevalent BCRs exhibited strong affinity for the H3K27M peptide rather than the H3 wt control, thus suggesting that the increased B‐cell population in the CSF was also triggered by the vaccine. They speculate that these elevated tumour‐reactive B cells could support the memory T‐cell population [13] and could potentially activate the complement system following the secretion of neoantigen‐specific antibodies, thereby indirectly promoting tumour clearance [14]. The observations by Boschert et al. align with previous findings indicating that B‐cell populations in lymphoid structures correlate with immunotherapy response [15, 16].

For TCRs to recognise and bind neoepitopes, they need to be first presented on the cell surface by the HLA system. To understand whether other patients would respond similarly to the patient described by Boschert et al., it is fundamental to consider the diversity of HLA alleles involved in this case and interpret their ability to present H3K27M‐derived epitopes. To explore this, the authors initially used CRISPR to disrupt the three major HLA II complexes (HLA‐DP, HLA‐DQ and HLA‐DR) in B cells (B‐LCL) derived from the patient, and observed a complete disruption of TCR reactivity as a result. By comparing the patient's B‐LCL with a panel of healthy donor B‐LCL, they pinpointed specific HLA alleles, ultimately demonstrating that their H3K27M peptide vaccine induces an immune response across diverse HLA alleleotypes. On the back of this, the authors confer that 40–45% of the German population, where the INTERCEPT‐H3 trial is ongoing, could experience a response mirroring that of the patient in this study. These observations ultimately suggest that their vaccine might be effective for a wide range of people, indicating potential benefits for populations worldwide.

The findings described by Boschert et al. provide valuable insights into the breadth and specificity of immune responses following therapeutic cancer vaccination, an aspect often overlooked in clinical studies of this kind. By analysing the immune response of a patient who showed exceptional response to the H3K27M peptide vaccine, the authors found CD4^+^ T cells as crucial drivers of this response, and pointed to mechanisms through which the T cells recognise and fight tumour cells. Furthermore, the group identified TCR and BCR sequences specific to the H3K27M target, thereby functionally validating the efficacy of their—and potentially other—peptide vaccinations as an effective personalised cancer therapy. These identified sequences could facilitate the engineering of genetically modified B and T cells, carrying these specific tumour‐targeting receptors, offering an alternative therapeutic strategy. The findings that diverse HLA loci can present the neoepitope expand the potential applicability of this vaccine strategy across different patient populations.

The investigation by Boschert et al. marks a significant advancement in understanding how peptide vaccines can induce robust immune responses in cancer therapy, particularly for cancers with specific mutations such as the H3K27M DMG in this study. The ongoing INTERCEPT‐H3 trial will provide further insights into the effectiveness of this vaccine. The outcomes of this trial will undoubtedly be of value for future therapies that harness the body's immune system to fight relentless tumours such as DMG and other difficult‐to‐treat cancers.

## Conflict of interest

The authors declare no conflict of interest.

## Author contributions

CAV and SR were involved in conceptualisation and literature analysis. CAV was involved in writing the original manuscript draft. SR was involved in reviewing, editing and writing the final manuscript. All authors have read and agreed to the published version of this article.
